# Value of ^18^F-FDG-PET to predict PD-L1 expression and outcomes of PD-1 inhibition therapy in human cancers

**DOI:** 10.1186/s40644-021-00381-y

**Published:** 2021-01-13

**Authors:** Kyoichi Kaira, Ichiei Kuji, Hiroshi Kagamu

**Affiliations:** 1 Department of Respiratory Medicine, Comprehensive Cancer Center, International Medical Center, Saitama Medical University, Saitama University Hospital, 1397-1 Yamane, Hidaka-City, Saitama, 350-1298 Japan; 2 Department of Nuclear Medicine, International Medical Center, Saitama Medical University, Saitama, Japan

**Keywords:** ^18^F-FDG-PET, Immunotherapy, Immune checkpoint inhibitor, Human neoplasm, PD-L1

## Abstract

Anti-programmed cell death-1 (PD-1)/programmed death ligand-1 (PD-L1) antibodies are administered in varied human cancer types. The expression of PD-L1 within tumor cells has been identified as a predictive marker, although assessing its expression has benefitted only patients with non-small cell lung cancer (NSCLC) or head and neck cancer. Whereas, more than 75% of the patients with NSCLC showing partial response to PD-1 blockade therapy experienced long-term survival for more than 5-years Thus, identifying the responders to PD-1 blockade at early phase after its initiation is of clinical importance. The 2-deoxy-2-[fluorine-18] fluoro-D-glucose (^18^F-FDG) on positron emission tomography (PET) can evaluate any tumor shrinkage by assessing the metabolic tumor volume at an earlier phase than conventional modalities such as computed tomography (CT). While several reports describe the correlation of PD-L1 expression with ^18^F-FDG uptake rate in the tumor cells, it remains to be delineated whether this rate determined by the glucose metabolism and hypoxia is associated with the status of immune microenvironment, including the expression of PD-L1. Moreover, details of the relationship between expression of PD-L1 and ^18^F-FDG uptake is still unclear. Therefore, we reviewed the clinical significance of ^18^F-FDG uptake on PET as a predictor of the efficacy of PD-1 blockade therapy, by correlating with the expression of PD-L1, in patients with several neoplasms.

## Introduction

Immune checkpoint inhibitors (ICIs) targeting programmed cell death-1 (PD-1) or programmed death ligand-1 (PD-L1) are administered to patients with varied tumor types. Although several novel ICIs are available, about 20% of the NSCLC patients who received anti-PD-1 antibody nivolumab showed low 5-year survival rate [1]. Moreover, the expression of PD-L1 in tumor cells has been described as predictor of response to therapy in patients with advanced NSCLC treated with anti-PD-1 pembrolizumab antibody [2]. However, apart from lung cancer or head and neck cancer [3, 4], the clinical significance of the PD-L1 expression as a predictive marker remains obscure. The tumor mutation burden (TMB) or peripheral blood mononuclear cells (PBMC) have been found useful for prediction of response to ICIs [5, 6]; however, except for PD-L1 expression by immunohistochemistry, biomarkers with better prognostic efficacy remain to be established.

Several recent studies have described the expression of PD-L1 within tumor cells to be closely associated with accumulation of 2-deoxy-2-[fluorine-18] fluoro-D-glucose (^18^F-FDG) in positron emission tomography (PET), replacing hypoxia inducible factor 1α (HIF-1α) as an alternative marker [7–15]. While the relationship between PD-L1 expression and the uptake of ^18^F-FDG in human neoplasms remains unclear, it is immunohistochemically supported by several studies [8–12]. The ^18^F-FDG-PET has been found useful for therapeutic monitoring after administration of systemic chemotherapy or molecular targeted therapy in patients with varied human cancer types, especially lung cancer [16–18]. However, only few reports describe the role of ^18^F-FDG-PET in therapeutic monitoring of anti-PD-1/PD-L1 antibodies. Our group has recently investigated the usefulness of ^18^F-FDG-PET in therapeutic evaluation at an early phase following initiation of nivolumab treatment in patients with previously treated NSCLC [19]. Our analysis suggested that ^18^F-FDG-PET potentially predicts precise therapeutic assessment at 1 month after nivolumab treatment, whereas, chest CT failed to differentiate a partial response from progressive disease at an early phase in the same duration. Furthermore, a prospective study is ongoing to confirm the results of our exploratory study [19].

Several reports from Western countries describe the relationship between uptake of ^18^F-FDG and ICIs or PD-L1 expression. Additionally, the correlation of ^18^F-FDG accumulation with PD-L1 expression within tumor cells reflects results similar to those obtained in previous investigation. Here, we have summarized the findings of previous reports to understand the potential of ^18^F-FDG-PET to predict the therapeutic potential of ICIs in human cancers.

### Relationship between FDG accumulation and PD-L1 expression

The uptake of ^18^F-FDG by tumor cells is closely associated with glucose metabolism and hypoxia [20]. The expression of glucose transporter 1 (GLUT1) for glucose metabolism and HIF-1α in response to hypoxia play a crucial role in the accumulation of ^18^F-FDG, and correlate with tumor progression and spread. While the expression levels of PD-L1 are related to the therapeutic efficacy of treatment with anti-PD-1 antibody [2], studies have correlated ^18^F-FDG uptake with PD-L1 expression [8–10]. Moreover, it has been reported that the expression of PD-L1 is associated with GLUT1 and HIF-1α expressions in patients with pulmonary pleomorphic carcinoma and renal cell carcinoma (RCC) [11, 12]. In this section, we reviewed the relationship between ^18^F-FDG uptake and PD-L1 expression in cancer cells from the viewpoint of basic, pathological, and clinical evidence.

#### Basic research aspect

With respect to the mechanism of ^18^F-FDG uptake, HIF-1α is an essential factor linked to the upregulated expression of GLUT1. A recent study indicated that the increased expression of HIF-1α is associated with the enhanced expression of PD-L1, and contributes to the activation of T-cell function and mitogen-activated protein kinase (MAPK) and phosphoinositide-3-kinase (PI3K) signaling pathways [14]. Furthermore, HIF-1α directly binds to the hypoxia response element in the proximal promoter of PD-L1 and controls its expression under hypoxia [15]. These results recommend PD-L1 as an alternative target of HIF-1α, and the rate of glucose metabolism determined by HIF-1α may reflect the immune response based on the expression of PD-L1. Barsoum et al. reported that the up-regulation in expression of PD-L1 in tumor cells upon exposure to hypoxia, in vitro, increased the rate of apoptosis of cytotoxic T lymphocytes, which suggests that HIF-1α plays a crucial role in driving immune escape from cytotoxic T lymphocytes, and that the inhibition of PD-L1 expression in hypoxic tumor cells could be promising for cancer immunotherapy [21]. Moreover, Tomita et al. examined the effect of anti-PD-1 antibody on ^18^F-FDG uptake in an immune activated tumor system using the cyclic dinucleotide GMP-AMP (cGAMP)-injected B16F10 melanoma model [22]. Their study indicated that administration of a PD-1 inhibitor increased the rate of infiltration of immune cells into tumors and resulted in significantly lower levels of GLUT1^high^ /hexokinase-II^high^ cells in CD45^−^ cancer cells, suggesting that the change in glucose uptake activity is associated with a difference in levels of infiltration or activation of immune cells. They emphasized that the tumor immune microenvironment, upon treatment with PD-1 inhibitor, affects the glucose metabolism in tumor cells and influences the uptake of ^18^F-FDG via factors such as the immune cell infiltration, activation and composition [22]. Moreover, recent investigations described that the direct blockade of the PD-L1 in cancer cells suppresses glycolysis by inhibiting mTOR activity and expression of glycolysis enzymes [23]. Although supported by a few studies, the association between the expression of PD-L1 and ^18^F-FDG uptake mandates further investigation.

#### Clinicopathological aspect

**Fig. 1:**
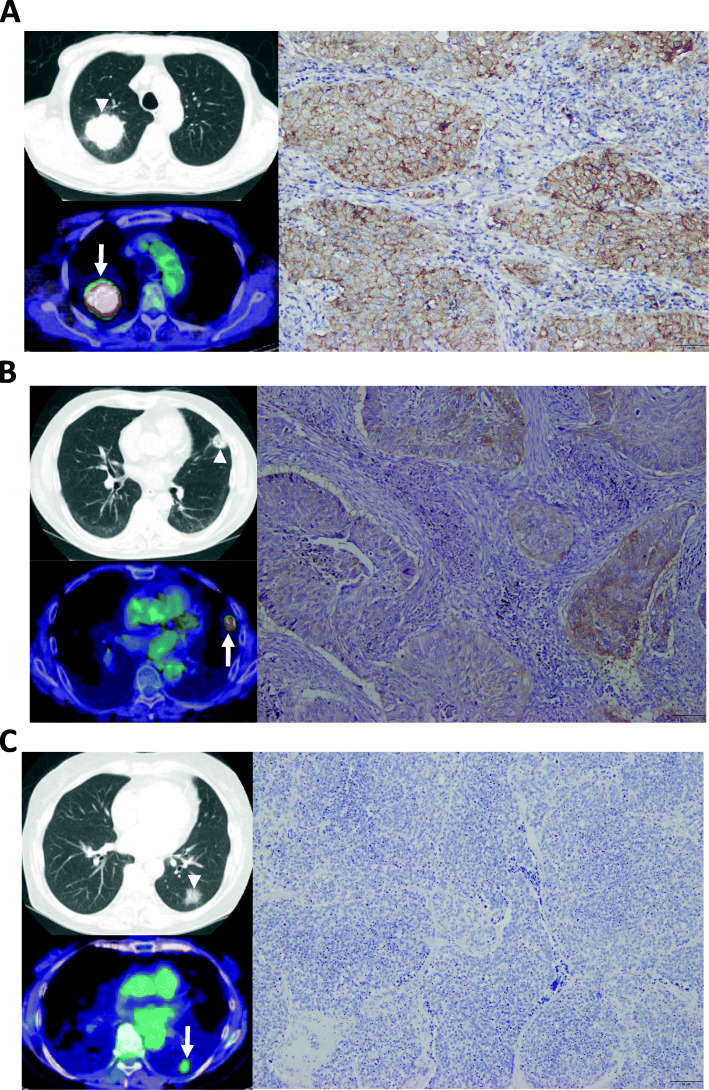
Different uptake of ^18^F-FDG on PET according to the expression level of PD-L1 within tumor cells. **a** 80-years old male with poorly differentiated adenocarcinoma (p-T2bN0M0); PET showed increased accumulation of ^18^F-FDG (white arrow) with SUV_max_ of 10.2 in the primary site corresponding to CT (white arrowhead). Immunohistochamical finding of this resected primary lesion exhibited high expression of PD-L1 with more than 50%. **b** 63-years old male with squamous cell carcinoma (p-T1bN0M0); moderate uptake of ^18^F-FDG (white arrow) with SUV_max_ of 5.6 was observed in the primary site corresponding to CT (white arrowhead). Moderate expression of PD-L1 with 1–49% was assessed in this resected primary site by immunohistochemistry. **c** 68-years female with well differentiated adenocarcinoma (p-T1bN0M0); PET revealed weak accumulation of ^18^F-FDG (white arrow) with SUV_max_ of 2.4 in the primary site corresponding to CT (white arrowhead). Immunohistochamical finding of this resected primary lesion displayed no expression of PD-L1 with less than 1%

Recent studies reported the positive correlation of PD-L1 expression with the uptake of ^18^F-FDG in patients with several human cancers, particularly lung cancer [7–10, 15, 24–30]. The relationship between PD-L1 expression and ^18^F-FDG uptake described in a review of literature is listed in Table 1. Of these 11 studies, seven were on lung cancer and four were on neoplasms originating in the colon and rectum, bladder, breast, and nasopharynx. We observed a significant correlation of ^18^F-FDG uptake with the expression of PD-L1 by immunohistochemistry, except in small cell lung cancer (SCLC) (Table 1). Patients with NSCLC also showed similar results, irrespective of the histological subtype or expression levels of PD-L1 [7–10, 25, 27]. Moreover, five studies performed immunohistochemical assessment of the correlation between ^18^F-FDG uptake and tumor infiltrative lymphocytes (TILs) [8, 9, 24, 27, 30]. While two studies indicated that the expression level of PD-L1 was not closely associated to the count of TILs, such as CD4, CD8, and Foxp3-regulatory T cells (Tregs) in NSCLC [8, 9], one study reported a statistically significant correlation between the maximum standardized uptake value (SUV_max_) and expression of CD8 TILs, CD163 tumor-associated macrophages, and Tregs [27]. Kasahara et al. demonstrated that a high SUV_max_ on ^18^F-FDG-PET significantly correlated with low expression of CD4 and CD8 TILs in patients with SCLC, but not with that of Tregs and PD-L1 [24]. Hirakata et al. also examined the relationship between ^18^F-FDG uptake and levels of PD-L1/TILs in patients with breast cancer, and their results indicated a significant association between SUV_max_ and levels of CD8 TILs, and SUV_max_ and expression of PD-L1. Based on these evidences, the relationship between accumulation of ^18^F-FDG and PD-L1 expression in tumor cells appears meagre. However, the association between ^18^F-FDG uptake and TILs appeared to be different according to the histological grade and cancer type. Therefore, further investigation is warranted to elucidate the clinicopathological significance of ^18^F-FDG uptake in PET based on the number of TILs in several human neoplasms. The ^18^F-FDG uptake on PET based on the expression of PD-L1 has been represented in Fig. 1.

**Table 1:** Relationship between PD-L1 expression and FDG uptake in review literatures

Authors/References	Sample size	Cancer type	Histology	Correlation between FDG uptake and PD-L1 expression		Correlation of FDG uptake with TILs (statistical assessment)
				***p***-value	PD-L1 clone	
Kasahara N / [8]	167	Lung cancer	SCC	0.02	E1L3N	Not significant
Kaira K / [9]	315	Lung cancer	AC	0.01	E1L3N/28–8	Not significant
Takada K / [7]	579	Lung cancer	SCC/AC/other	< 0.001	SP142	NA
Zhang M / [10]	84	Lung cancer	SCC	0.035	28–8	NA
Kasahara N / [24]	98	Lung cancer	SCLC	0.36	E1L3N	Significant
Hu B / [25]	362	Lung cancer	SCC/AC	0.001	28–8	NA
Wang Y / [27]	122	Lung cancer	SCC/AC	0.012	NA	Significant
Jiang H / [26]	65	Colon cancer	AC	0.000	28–8	NA
Chen R / [15]	63	Bladder cancer	UC/SCC/SRC	0.032	NA	NA
Zhao L / [28]	84	NPC	SCC	< 0.001	SP263	NA
Hirakata T / [30]	97	Breast cancer	AC	< 0.001	28–8	Significant

*Abbreviations*: *PD-L1* programmed death ligand-1, ^*18*^*F-FDG* 2-Deoxy-2-[^18^F] fluoro-D-glucose, *TILs* tumor infiltrative lymphocytes, *SCC* squamous cell carcinoma, *AC* adenocarcinoma, *SCLC* small cell lung cancer, *UC* urothelial cancer, *SRC* signet ring cell carcinoma, *NPC* nasopharyngeal carcinoma, *NA* not applicable

The SUV_max_ is clinically utilized for evaluating the metabolic levels of ^18^F-FDG within the tumor cells. While SUV_max_ actually reflects the maximum extent of glucose metabolism within tumor cells, whether it can represent the complete metabolic tumor volume remains unclear. A recent meta-analysis found that metabolic tumor volume (MTV) and total lesion glycolysis (TLG) are better predictive markers of ^18^F-FDG uptake than SUV_max_ [31]. In previous studies, it has been reported that the expression of PD-L1 was significantly correlated with the uptake of ^18^F-FDG by not only SUV_max_ but also TLG or MTV. However, it remains unclear whether the correlation of PD-L1 expression with SUV_max_ is stronger than that with TLG or MTV.

### ^18^F-FDG-PET in therapeutic monitoring of immunotherapy

The degree of effectiveness of ^18^F-FDG-PET to differentiate responders from non-responders at the early phase after administration of ICIs remains unclear. However, several reports are available regarding the usefulness of ^18^F-FDG-PET for the therapeutic monitoring of immunotherapy in patients with several human neoplasms, particularly NSCLC and malignant melanoma. In this section, we reviewed the clinical significance of ^18^F-FDG-PET for response evaluation before and after treatment with ICIs according to cancer types.

#### Malignant melanoma

The time-point for assessing the therapeutic response based on the uptake of ^18^F-FDG on PET before or after ICI administration remains to be validated. Seith et al. examined the potential of ^18^F-FDG on PET to identify complete responders to PD-1 therapy (nivolumab or pembrolizumab) at 2 weeks after its initiation in patients with metastatic melanoma [32]. They prospectively recruited 10 patients who underwent ^18^F-FDG-PET scan at three time points–before start of therapy, and two weeks and three months after initiation of therapy. Of these, three patients showed a partial metabolic response (PMR) at 2 weeks using ^18^F-FDG-PET and confirmed complete metabolic response (CMR) after 3 months. Four patients with progressive metabolic response (PMD) at 2 weeks exhibited the same response after 3 months. The results of their preliminary study suggested that ^18^F-FDG-PET could detect complete responders to anti-PD-1 therapy as early as 2 weeks after initiation of treatment for advanced melanoma. Further, Cho et al. prospectively investigated the prediction of response to ICIs (ipilimumab, BMS-936559, and nivolumab administered in 16, 3, and 1 patient, respectively) using ^18^F-FDG-PET before initiation of therapy, at days 21–28 and at 4 months [33] in 20 patients with advanced melanoma. Response evaluations performed at 21–28 days using response evaluation criteria in solid tumors (RECIST) by CT and PET evaluation criteria in solid tumors (PERCIST) by ^18^F-FDG-PET exhibited 75 and 70% accuracy in predicting best overall response at more than 4 months, respectively. Their study described that the combination of functional (^18^F-FDG-PET) and anatomical (CT) imaging at an early phase after treatment with ICIs improved the predictive potential of response to ICIs with 100% sensitivity, 93% specificity, and 95% accuracy. Additionally, several retrospective studies have elucidated the clinical predictive potential of ^18^F-FDG-PET [34, 35]. Annovazzi et al. observed that ^18^F-FDG-PET scan at 3–4 months after treatment with ICIs could accurately indicate response to treatment and predicted long-term outcome in 57 patients with metastatic melanoma (25 patients received ipilimumab and 32 patients received PD-1 inhibitors) [34]. Furthermore, a recent retrospective analysis of metastatic melanoma patients (*n* = 104) treated by PD-1 inhibitors demonstrated that most patients with a partial response (PR) based on the RECIST achieved CMR in ^18^F-FDG-PET at 1 year after initiation of treatment with a PD-1 inhibitor, and the majority of patients with CMR at 1 year exhibited continued response to treatment thereafter, suggesting the clinical utility of ^18^F-FDG-PET in predicting the long-term survival [35].

#### Non-small cell lung cancer

Our group prospectively investigated the therapeutic monitoring of ^18^F-FDG-PET as a predictive marker of early response after administration of nivolumab in 24 patients with previously treated NSCLC [19]. The results indicated significant efficacy of ^18^F-FDG uptake determined by TLG to predict the probability of a partial response (PR) (100% vs. 29%) and progressive disease (PD) (100% vs. 22.2%) at 1 month after treatment with nivolumab than that predicted by CT scans. Moreover, TLG and MTV were found to be better in appropriately assessing the metabolic activity than SUV_max_ by ^18^F-FDG uptake. A multi-institutional prospective study is currently ongoing to test the results of our preliminary study (jRCTs031180036). Further, Humbert et al. have reported the therapeutic significance of ^18^F-FDG-PET in the early assessment of anti-PD-1 immunotherapy (pembrolizumab and nivolumab) in patients (*n* = 50) with NSCLC [36], which was evaluated according to three points—at baseline, and after 7 weeks and 3 months of treatment. The analysis according to PERCIST, based on SUV_max_, indicated a durable clinical benefit at 7 weeks after the treatment with 85.7% sensitivity, 62.1% specificity, and 72.0% accuracy. They also speculated that subsequent PET would be useful to identify more than 50% of the patients with an atypical response pattern among those with prior PD based on PERCIST. Next, a retrospective analysis of 28 NSCLC patients who received nivolumab evaluated the therapeutic assessment of ^18^F-FDG-PET before and after 2 months of treatment [37]. The sequential PET scan indicated CMR and PMR in 11 patients after 2 months of treatment, and 13 patients were identified with PMD, of whom 9 (69%) were confirmed as non-responders. In these three studies, the patients with PR on ^18^F-FDG-PET were found to be associated with favorable prognosis after immunotherapy [19, 36, 37], even though MTV and TLG were utilized in only one study [19]. Additionally, the potential of ^18^F-FDG-PET to predict the major pathological response (MPR) upon treatment with neoadjuvant anti-PD-1 antibody (sintilimab) was examined in 36 patients with resectable NSCLC [38]. The study indicated a significant correlation between the MPR and PET response based on PERCIST, and all the patients (100%) with PMR tumors were found to exhibit MPR, indicating the usefulness of ^18^F-FDG-PET in predicting the MPR to the neoadjuvant PD-1 blockade.

**Fig. 2:**
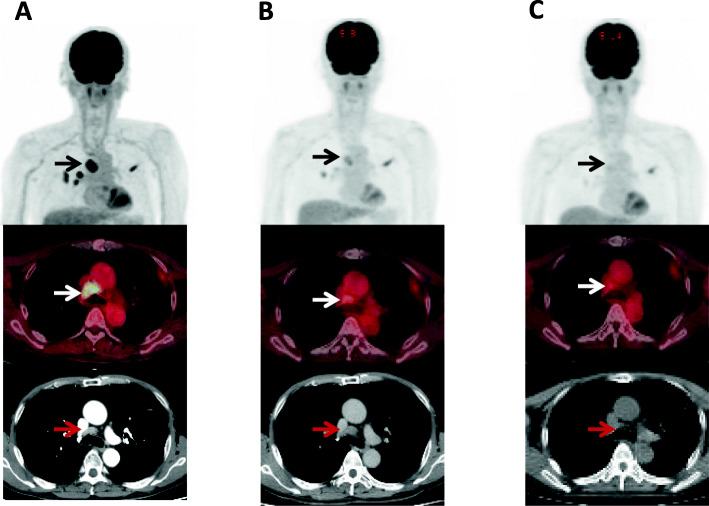
Therapeutic monitoring of ^18^F-FDG-PET in 71-years old male with advanced pulmonary adenocarcinoma who received pembrolizumab monotherapy as 1st line treatment. **a** ^18^F-FDG-PET/CT before pembrolizumab initiation shows primary site in the right upper lobe and hilum-mediastinal lymphadenopathy. Mediastinal lymphadenopathy before trachea revealed increased accumulation of ^18^F-FDG (black and white arrows) with SUV_max_ of 15, corresponding to that on CT scan (red arrow). **b** At 1 month after pmebrolizumab treatment, uptake of ^18^F-FDG on PET was obviously decreased with SUV_max_ of 3, but the morphological size of its lymphadenopathy was not changing (red arrow). **c** At 3 months after its treatment, there was no accumulation of ^18^F-FDG in primary site and lymphadenopathy (black and white arrows) with morphological shrinkage on CT scan (red arrow)

**Fig. 3:**
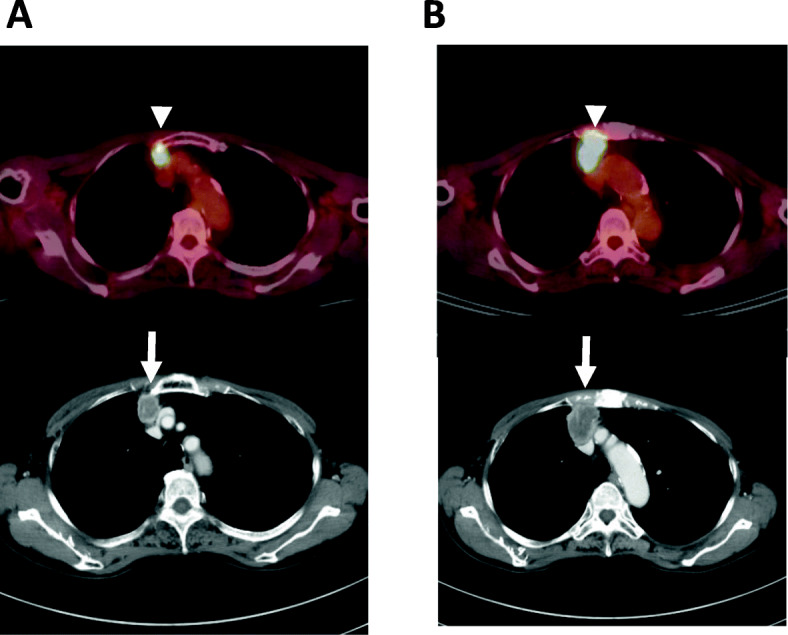
Imaging of ^18^F-FDG-PET in 74-years old female with advanced pulmonary adenocarcinoma who was treated with nivolumab monotherapy as 3rd line treatment. **a** PET imaging displayed the increased accumulation of ^18^F-FDG with SUV_max_ of 5.2 (white arrowhead) in the anterior mediastinal lymph node metastasis corresponding to CT (white arrow). **b** At 1 month after nivolumab initiation, the growth of the lymph node metastasis was observed on CT scan (white arrow) with increased uptake of ^18^F-FDG with SUV_max_ of 11.3 (white arrowhead)

Next, several retrospective studies have described the prognostic significance of ^18^F-FDG uptake on PET before initiating treatment with PD-1 inhibitors [39–41]. Hashimoto et al. retrospectively investigated the prognostic significance of ^18^F-FDG uptake as a predictive marker before treatment with anti-PD-1 antibody in 85 patients with previously treated NSCLC [39]. The tumor metabolic activity assessed by TLG and MTV, but not SUV_max_, was confirmed as a significant independent prognostic factor to predict the prognosis after treatment with PD-1 inhibitor based on the multivariate analysis [39]. Furthermore, in 109 patients with advanced NSCLC who underwent a baseline ^18^F-FDG-PET scan before the ICI monotherapy, MTV on PET was found to be closely associated with a worse outcome and absence of a durable clinical benefit, unlike SUV_max_ [40]. However, Takada et al. examined the clinical significance of pretreatment ^18^F-FDG-PET in recurrent patients (*n* = 89) treated with anti-PD-1 antibody, and the analysis indicated the average SUV_max_ of the responders to be significantly higher than that of the non-responders [41]. These results support the utility of SUV_max_ on PET as a potential predictor of therapeutic response to PD-1 inhibitors. Although several reports have suggested the predictive potential of ^18^F-FDG uptake, it remains unclear whether the assessment of ^18^F-FDG uptake based on MTV or TLG can better predict the outcome than SUV_max_ after initiation of treatment with ICIs. ^18^F-FDG-PET scan images depicting the patients’ response to the PD-1 inhibitor are shown in Figs. 2 and 3.

#### Other malignancies

In nine patients with metastatic RCC who received nivolumab, ^18^F-FDG-PET was used to assess the early response at baseline and at 1 month after treatment [42]. The analysis confirmed that elevation in the uptake of ^18^F-FDG at 1 month was an independent predictor among therapy responders based on a multivariate logistic regression analysis. Furthermore, two studies reported that SUV_max_ and MTV on ^18^F-FDG uptake at 2 or 3 months after treatment with anti-PD-1 antibodies predict the outcome and response in patients with Hodgkin lymphoma [43, 44]. Moreover, Ferdinandus et al evaluated the PET response and survival of 27 patients with malignant mesothelioma who received ^18^F-FDG-PET at baseline and after at least 4 cycles pembrolizumab, and found that ^18^F-FDG PET metabolic volume response could predict outcome for such patients [45].

## Discussion

Here, based on results of several studies, the expression of PD-L1 was found to be closely associated with the uptake of ^18^F-FDG on PET in different cancer types. As indicated in the basic research studies, PD-L1 was found to be a potential alternative marker of HIF-1. Moreover, the tumor glucose transporter and hypoxia were observed to be related to the expression of PD-L1 based on the immunohistochemical analysis. The aim of this review was to evaluate whether the levels of uptake of ^18^F-FDG could be useful for therapeutic monitoring of anti-PD-1/PD-L1 antibodies. However, the differences in the timing of ^18^F-FDG-PET after initiation of treatment with ICIs in each study make it difficult to predict their therapeutic efficacy. A morphological assessment using CT scan can predict the response of PD-1 inhibitor at approximately 9 weeks after its initiation. Although, since more than half of the patients who receive PD-1 inhibitor experience progressive disease at 9 weeks after its initiation, the responders should be distinguished from non-responders at early phase, such as 2 or 4 weeks after the treatment. Furthermore, two studies [19, 31] indicated that ^18^F-FDG-PET performed at 2 or 4 weeks after treatment with a PD-1 inhibitor permitted an early detection of responders and non-responders. Thus, these results should be validated using prospective studies to test whether the uptake level of ^18^F-FDG could accurately predict the efficacy at an early phase (2 or 4 weeks) after treatment with a PD-1 inhibitor.

In a recent analysis, Niemeijer et al. demonstrated that the expression of PD-1 and PD-L1 can be quantified using PET in patients with NSCLC [46]. They confirmed the safety and feasibility of in vivo molecular imaging of PD-1/PD-L1 using ^18^F-BMS-986192 and ^89^Zn-nivolumab; the tumor accumulation of ^18^F-BMS-986192 correlated with PD-L1 expression, while the uptake of ^89^Zn-nivolumab correlated with the expression of PD-1. The results of this study suggest that these PET tracers can be used to quantify the expression of PD-L1/PD-1 in studies with ICIs. Moreover, Bensch et al. reported the potential of ^89^Zn-atezolizumab PET imaging to assess the clinical response to PD-L1 blockade in first-in human cancer [47]. In their study, the tracer uptake of ^89^Zr-aterzolizumab was identified as a predictor of the response and outcome upon treatment with aterzolizumab. Thus, the PD-1/PD-L1 labeled PET tracer could serve as potential response predictor for PD-1 blockade therapy.

## Conclusions

Although precise mechanism of how accumulation of ^18^F-FDG on PET correlates with expression of PD-L1 remains to be elucidated, the results of several independent studies are consistent irrespective of the cancer types. The uptake of ^18^F-FDG at an early phase after initiation of PD-1 blockade therapy could aid in therapeutic monitoring. However, the optimal timing of post-treatment ^18^F-FDG-PET is still obscure. Thus, further large-scale prospective studies are warranted to elucidate the novel possibility of therapeutic monitoring with ^18^F-FDG-PET after initiation of PD-1 inhibitor therapy.

## Data Availability

Not applicable for a review paper.

## Abbreviations

ICIs: Immune checkpoint inhibitors
PD-1: Programmed cell death-1
PD-L1: Programmed death ligand-1
NSCLC: Non-small cell lung cancer
TMB: Tumor mutation burden
PBMC: Peripheral blood mononuclear cell
^18^F-FDG: 2-deoxy-2-[fluorine-18] fluoro-D-glucose
PET: Positron emission tomography
CT: Computed tomography
GLUT1: Glucose transporter 1
HIF-1α: Hypoxia inducible factor-1α
RCC: Renal cell carcinoma
MAPK: Mitogen-activated protein kinase
PI3K: Phosphoinositide-3-kinase
cGAMP: Cyclic dinucleotide GMP-AMP
TILs: Tumor infiltrative lymphocytes
Tregs: Foxp3-regulatory T cells
SUV_max_: Maximum standardized uptake value
MTV: Metabolic tumor volume
TLG: Total lesion glycolysis
PMR: Partial metabolic response
CMR: Complete metabolic response
PMD: Progressive metabolic response
PERCIST: PET evaluation criteria in solid tumors
RECIST: Response evaluation criteria in solid tumors
PR: Partial response
PD: Progressive disease
MPR: Major pathological response
